# ‘Taking Sustainable Food Systems Teaching Out of the Box’: Embedding Transformative, Systems‐Engaged Pedagogy in Canadian Dietetic Education

**DOI:** 10.1111/jhn.70187

**Published:** 2025-12-28

**Authors:** Jessica Wegener, Liesel Carlsson, Tracy Everitt, Naomi Kereliuk, Jacqueline Stokes

**Affiliations:** ^1^ School of Nutrition Toronto Metropolitan University Toronto Ontario Canada; ^2^ School of Nutrition and Dietetics Acadia University Wolfville Nova Scotia Canada; ^3^ Department of Human Nutrition St. Francis Xavier University Antigonish Nova Scotia Canada

**Keywords:** curriculum, dietetics, education, pedagogy, sustainable food systems, systems thinking

## Abstract

**Purpose:**

Canadian dietetic educators are incorporating sustainable food systems (SFS) education into teaching and training as a goal towards more systems‐oriented practice. This research examines SFS education in accredited dietetics programs within a Canadian post‐secondary institution. The purpose is to examine how SFS is covered and to identify barriers, resource needs, and required supports among dietetic educators.

**Methods:**

A cross‐sectional, mixed‐methods survey design consisting of two phases was used. A convenience sample of dietetic educators who had taught an SFS‐related course within the past 5 years completed a web‐based questionnaire (Phase 1) and were invited to participate in an in‐depth interview (Phase 2). Quantitative data were analyzed using descriptive statistics and qualitative responses were analyzed thematically.

**Results:**

Sixteen dietetic educators, representing 11 eligible institutions, participated in the study. Five participants took part in an interview. Despite widespread support for SFS, SFS education is largely limited to one course per program. Several integrated challenges concerning limited SFS coverage were identified, including a lack of coordinated, scaffolded learning, a minimum curriculum that is instructor‐dependent, and the need for pedagogy that supports effective learning. Despite a clearly recognized need for support from departments, institutions and professional associations, promising examples of SFS teaching were identified.

**Conclusions:**

To equip future professionals with the knowledge and skills necessary to facilitate healthy and sustainable dietary patterns and food environments, coordinated action is needed. Program directors can signal the importance of SFS by incorporating it into the curriculum and intentionally scaffolding SFS across multiple program courses. Professional regulators can enhance the specificity of SFS within the competency standards. University administrators can lead within the institution, signalling the importance of SFS and creating opportunities for interdisciplinary teaching and learning. These findings can be used to better support programs and plan for more intentional, multi‐year, transformative and systems‐engaged SFS pedagogy.

## Summary

- Dietetic educators can advocate for specific sustainable food systems (SFS) concepts in the standards governing the content of accredited dietetics programs to help facilitate systemic integration of SFS education, impacting all programs.
- Dietetic educators can develop resources and tools for consistent training and utilize the growing collection of lesson plans, online resources and resource banks available (described within this paper) to promote standardized preparedness among dietetic program graduates.
- To foster systems thinking, dietetic programs should aim to ‘unbox’ SFS content from a single course or module and strategically integrate key SFS learning across multiple courses in upper and lower program years in a scaffolded approach.

## 1 Introduction

### 1.1 Integrating Sustainable Food Systems into Dietetics Education

Among a growing number of national dietetic associations, Dietitians of Canada (DC) recognizes the inseparable relationship between sustainable food systems (SFS) and human health [1, 2]. This recognition, supported broadly at a global level (e.g., the EAT‐Lancet Commission), suggests that healthy diets are closely tied to societal and planetary health. Dietitians are well‐positioned to support transitions to healthy, sustainable diets and food environments because they have influence in many areas of food and health systems [3, 4]. The integration of SFS education within dietetic education and training is both a professional obligation and a means to strengthen the impact of dietitians on planetary health. Although many complex factors influence the consumption of a sustainable diet [5], healthy, sustainable dietary patterns promote all dimensions of health and well‐being; have low environmental pressure and impact; are accessible, affordable, safe, and equitable; and are culturally acceptable [6]. Despite decades of awareness regarding the link between food systems and ecological sustainability [7, 8, 9], the integration of SFS into dietetics education is inconsistent and lacks systemic integration due to limited faculty training, a lack of resources, and curriculum constraints [10, 11]. However, given a growing number of challenges posed by our current food systems on our ecological (e.g., climate change) and social systems (e.g., food insecurity, human nutrition), there is a need to equip dietitians with the theoretical knowledge and practical skills to advocate and promote SFS [8, 12, 13].

### 1.2 Embedding Sustainability in Dietetics Curricula

Universities play a crucial role in food system transformation by integrating sustainability principles into their curricula [14, 15, 16, 17, 18] and fostering leadership among nutrition professionals [13]. Students overwhelmingly support the integration of sustainability, yet report limited familiarity with core concepts, highlighting the need for structured curricular enhancements [19, 20]. Evidence suggests that embedding sustainability across multiple courses, rather than as a standalone course, fosters systems thinking and enhances long‐term competency development [16]. This scaffolded approach, utilizing consistent language and providing reinforced learning opportunities across courses and program years, promotes deeper engagement with sustainability issues [19, 21]. Short‐term educational interventions have demonstrated limited efficacy in shaping sustainability‐oriented professional behaviours [22]. In contrast, longitudinal exposure to sustainability concepts through structured coursework, experiential learning, and interdisciplinary collaboration has been shown to enhance the retention and application of competencies [16, 23]. Sustainability‐focused practicums, problem‐based learning, and collaborative projects offer students opportunities to critically engage with complex environmental issues, thereby reinforcing their role in advancing SFS [24].

### 1.3 Dietetic Accreditation Standards

The integration of SFS into dietetics education is driven by ethical imperatives, student demand, and professional accreditation standards [20, 22, 24, 25]. Situating the promotion of healthy diets and food environments as part of socially and ecologically SFS signals a new role and unique challenge for dietitians in meeting the health needs of all people living in Canada. This is a new way of framing dietetics work that dietitians often feel ill‐equipped to deliver in practice, let alone teach to dietetic trainees [13, 26].

The Canadian dietetic training standards, updated in 2020, incorporate SFS concepts into education and training [27]; however, they have been described as too general, requiring a lower level of cognitive complexity and offering inadequate guidance for SFS educators [28]. In contrast, the Revised 2020 (Voluntary) Standards of Professional Performance for Registered Dietitian Nutritionists in the United States are specific and provide a model that can be adapted to various education and training environments (across countries and regions), reinforcing the importance of sustainable food and water systems within dietetics [29].

The concept of civic dietetics, introduced by Wilkins et al. [8], highlights the need for a systems‐thinking approach that incorporates social justice and equity considerations into dietetic practice. Critical thinking and problem‐solving skills are essential for navigating the complexities of sustainability and food system transformation [25]. Equity remains an underrepresented component of sustainability education in North American dietetics programs. Many universities lack explicit curricular references to systemic oppression and food justice [30]. To address this gap, scholars advocate for competency models that incorporate strategies for dismantling inequities and fostering inclusivity in food systems [31, 32].

### 1.4 State of SFS‐Related Education and Training Practice in Canada

This study builds on a published international review of sustainability education in dietetic and nutrition programs in multiple countries [11]. Using a sustainability metric to assess course descriptions across 23 programs (18 undergraduate and five graduate), only eight Canadian courses referenced sustainability or SFS based on available descriptions. Of these, two considered sustainability alone (without a reference to food systems); four (6%) were offered as a single undergraduate lecture or topic; and only two were dedicated to SFS. Building on our prior knowledge of limited SFS education in Canada, the purpose of this research was to understand the current state of SFS‐related content in education theory and practice, including the perceived teaching barriers and needs among educators. The following research questions guided the study: (i) How are SFS concepts taught? (ii) What gaps exist related to SFS? (iii) Are there examples of effective SFS‐related education? and (iv) What supports are required by educators to ensure Canadian dietitians are prepared to play leadership roles facilitating healthy and sustainable dietary patterns and food environments?

## 2 Methods

### 2.1 Study Design

The research team employed a cross‐sectional, mixed‐methods survey design consisting of a web‐based questionnaire (Phase 1) that informed follow‐up through in‐depth interviews (Phase 2). Ethics approval was obtained through Toronto Metropolitan University (REB 2022‐316), Acadia University (REB 22‐41), and St. Francis Xavier University (REB 26076). The research team consisted of three researchers with over 20 years of experience as dietetic educators and registered dietitians (RD) representing three accredited dietetics programs in Canada. Each researcher brings their own positionality, shaped by the Canadian context and Western education, training and teaching experiences. These influence this research through preconceived ideas about sustainability and food systems, as well as interpretations of the data.

### 2.2 Sampling, Participants, and Recruitment

A purposive sampling strategy targeted representatives from each of Canada's accredited dietetics programs. The goal was not to obtain a representative sample of Canadian educators but rather to recruit those who had already begun to incorporate SFS into teaching. Based on an official program list (Partnership for Dietetic Education and Practice (PDEP), no date) at the time of this study, there were 28 accredited dietetics programs. The sampling strategy was designed and revised to recruit at least one representative from each of the post‐secondary institutions (*n* = 17) with an accredited dietetics program and knowledge of SFS education.

Eligible participants included dietetic educators and individuals involved in teaching an undergraduate or graduate course within the past 5 years within a program identified as part of the preparatory pathway to becoming an RD in Canada. Guest lecturers were not eligible to participate. Individuals were invited to participate through an emailed letter sent in January 2023 and recruited via professional networks, program websites, and snowball sampling. The letter included detailed information, and by clicking the link to initiate the survey and providing demographic information, participants indicated their consent.

### 2.3 Procedures

#### 2.3.1 Phase 1 Web‐Based Questionnaire

LimeSurveys software was used to host the English and French versions of the web‐based questionnaire and collate the managed open‐ and closed‐ended data. Questions about participant characteristics included institution, program, position, RD status and SFS‐related teaching within the last 5 years. Questions about teaching included content, barriers, supports, course name/code, delivery mode, level, mandatory/elective status, estimated percentage of course content dedicated to SFS concepts, learning objectives, use of a systems thinking approach, and the framework(s) and/or definition(s) used. Participants were invited to upload a copy of their course syllabus for further analysis.

All study materials were translated into French and reviewed by a French RD with research experience in SFS. To ensure content validity and reliability, the instruments were pilot‐tested with two dietetic educators, who did not participate in the study, to ensure the process and questions were aligned with the research questions being explored. Minor changes were made to the web‐based questionnaire and interview guide.

#### 2.3.2 Phase 2 Interviews

The purpose of the interviews was to explore educators' in‐depth perceptions of SFS coverage within their programs, including barriers, departmental and institutional support for SFS, other forms of support, and student needs and learning outcomes. In addition to those who completed the survey, participants were recruited for an interview via snowball sampling (i.e., potential interviewees were identified by respondents who had completed the survey on behalf of their program/institution). Potential interviewees were assessed for eligibility by a member of the research team using the same screening questions from the survey, which were used to determine program accreditation and influence over the education and training of students from a dietetic pathway program.

The interviews took place between October 2023 and June 2024 via *Zoom* (Version 6.0). Two members of the research team led the interviews using an English semi‐structured interview guide, and a research assistant (N.K.) took notes. The interview guide was populated with course details provided by the respondent on their web‐based survey. The interviews lasted approximately 60 min and were recorded and transcribed using Zoom's recording and transcription features. A second research assistant (J.S.) reviewed and compared the Zoom‐generated transcripts with the recordings, corrected inaccuracies, translated all French responses into English, and added additional notes.

### 2.4 Data Analysis

Quantitative survey data were analyzed by N.K. and J.S. using descriptive statistics, supported by Microsoft Excel. NVIVO software (Version 14) and Google Jam board were used to support the analysis of qualitative data from the transcribed interviews and open‐ended survey questions. The interview transcripts were de‐identified and uploaded to NVIVO for data management. J.W. conducted the initial analysis through active reading and re‐reading of the transcripts and survey responses and generated initial codes through line‐by‐line coding of the data using an inductive approach [33, 34]. J.W. proposed a coding template for L.C. and T.E. to use for independent coding. J.W., L.C., and T.E. reconvened to compare the independent coding, group similar codes into potential themes, and to identify and discuss relationships and connections within the coded data as they related to dietetic educators' perceptions of the gaps and supports needed for teaching SFS. Themes and subthemes were identified, described, deliberated and revised as needed until a consensus was reached on the final thematic analysis.

For participants who shared their course syllabi, N.K. systematically reviewed the documents to extract pertinent study information. The learning objectives of the SFS‐related courses taught by participants were analyzed using NVIVO's keyword query. The top 10 keywords used in Canadian course learning objectives are summarized in Table 2.

## 3 Results

### 3.1 Participant Characteristics

Sixteen participants, representing 11 (65%) Canadian post‐secondary institutions offering an eligible dietetics program, participated in at least one phase of the research. Fourteen respondents completed the web‐based survey, and three of them participated in an interview. Two participants, recruited through snowball sampling and screened for eligibility, participated in an interview but did not complete the survey. As shown in Table 1, study participants described 17 SFS‐related courses, with most (*n* = 14, 82%) offered at the undergraduate level. Participants were full‐time faculty (*n* = 9, 56%), part‐time instructors (*n* = 2, 12%) and staff educators or program coordinators (*n* = 5, 29%). The majority were RDs (*n* = 13, 81%). In total, there were five interview participants representing a unique program and institution.

**Table 1:** Participant characteristics.

Participant characteristics	Details	Number (%)
Institution (affiliation) (undergraduate and graduate dietetic programs) (*n*‐11)	University of British Columbia Universite de Montréal Mount Saint Vincent University University of Guelph University of Manitoba Toronto Metropolitan University University of Alberta University of Saskatchewan St. Francis Xavier University University of Toronto^a^ Memorial University of Newfoundland, Master of Public Health with Nutrition/Dietetics stream^a^	*n* = 2, 18% *n* = 3, 27% *n* = 1, 9% *n* = 1, 9% *n* = 1, 9% *n* = 2, 18% *n* = 1, 9% *n* = 1, 9% *n *= 2, 18% *n* = 1, 9% *n* = 1, 9%
Position/Job title	Full‐time faculty Part‐time/sessional faculty Other (staff educators, practicum coordinators)	*n* = 9 (56%) *n* = 2 (12%) *n* = 5 (29%)
Registered Dietitian	Yes (*n* = 13) No (*n* = 3)	*n* = 13 (81%) *n* = 3 (19%)
Number of SFS‐related courses	Instructing one course Instructing two courses	*n* = 15 (94%) *n* = 1 (6%)
Program type for courses taught	Undergraduate (*n* = 14)^b^ (82%) Graduate (*n* = 3) (18%)	*n* = 14 (82%) *n* = 3 (18%)
Number of years of incorporating SFS into participants' course(s):	< 1 year 1–2 years 3–4 years 5–6 years > 6 years Non‐response	0 *n* = 4 (25%) *n* = 1 (6%) 0 0 *n* = 11 (69%)

a Academic institution without an accredited dietetics program but with an option for practical training through a hospital partner (not counted within the 17 institutions with accredited dietetics programs).

b One survey respondent provided details on two SFS‐related courses on the web‐based survey and was counted as one study participant.

### 3.2 Overview of SFS‐Related Courses

Overall, SFS is taught as a single university course in more than half (64%) of the undergraduate dietetic programs in our study and in all three graduate programs. Three undergraduate programs offer more than one SFS‐related course. Graduate SFS‐related courses were consistently mandatory, whereas only 50% of the SFS‐related undergraduate courses, as described by participants, were mandatory. Approximately 43% of the courses described by participants were estimated to dedicate more than 50% of the course to SFS concepts (average: 66%) and introduce one or more dimensions of sustainability (environmental, social, or economic). However, there is a high degree of variability in the estimated SFS content coverage: undergraduate courses ranged from < 10% to> 90%, while graduate courses ranged from 8% to 70%.

The terms used in course titles, calendar descriptions, and course objectives vary widely. Some course descriptors indicate clear SFS coverage, with title terms such as ‘food systems,’ ‘ecology,’ ‘availability,’ and ‘land,’ as do course objective terms like ‘food systems,’ ‘sustainability,’ ‘security,’ and ‘sovereignty.’ Other terms are less direct, and SFS‐related content is taught under the banner of nutrition policy and community nutrition (as noted in course titles). Some terms emphasize the inclusion of values and equity, such as social justice, health equity, Indigenous food sovereignty, and ethical practice (as noted under course learning objectives).

Educators use a variety of frameworks and resources; however, none are consistently used or common across programs, except for the DC Role Paper and the 2019 EAT‐Lancet Commission report (see Table 2). Some topics were more challenging for educators to teach than others, especially topics related to professional roles and sustainability (e.g., sustainability/nutrition policies). Teaching about food waste, equity, gender, culture, and water use was comparably less challenging.

**Table 2:** Sustainable food system course details (*n* = 17).

	Undergraduate courses (*n* = 14)	Number (%)	Graduate courses (*n* = 3)	Number (%)
Word descriptions	food/nutrition policy; community nutrition; food and systems; introduction to food systems; land and food/ecology/availability (across all SFS‐related course titles)			
Course Delivery (Select all that apply)	In person: Experiential: Online synchronous: Service learning: Land‐based:	*n* = 8, 57% *n* = 4, 29% *n* = 3, 21% *n* = 2, 14%	In person: Experiential: Online synchronous: Service learning: Land‐based:	*n* = 3, 100%
Mandatory vs Elective	Mandatory: Elective: No response:	*n* = 7, 50% *n* = 9, 64%	Mandatory: Elective: No response:	*n* = 3, 100%
Year of Study	1st year (undergrad): 2nd year (undergrad): 3rd year (undergrad): 4th year (undergrad): No response (undergrad):	*n* = 1, 7% *n* = 1, 7% *n* = 2, 14% *n* = 1, 7% *n* = 9, 64%	n/a	
Approach to teaching and learning in the course	Lecture: Small/large group discussions of prior lecture/reading: Case studies: Project‐based learning: Community service learning: Interdisciplinary project learning with students from other disciplines: Other: e.g. food science labs; extra credit opportunities, tutorial sessions hosted by a teaching assistant, videos, games	*n* = 7, 50% *n* = 6, 43% *n* = 3, 21% *n* = 4, 29% *n* = 5; 36% *n* = 1, 7%	Lecture: Small/large group discussions of prior lecture/reading: Case studies: Project‐based learning: Community service learning: Interdisciplinary project learning with students from other disciplines	*n* = 2, 66% *n* = 3, 100% *n* = 3, 100% *n* = 1, 33%
Assessment tasks to evaluate student learning	Knowledge testing, such as true/false, multiple choice, and matching (**Knows**):	*n* = 6; 43%	Knowledge testing, such as true/false, multiple choice, and matching (**Knows**):	—
	Interpretation and application activities such as presentations, essays, literature reviews and analyses (**Knows How**):	*n* = 6, 43%	Interpretation and application activities such as presentations, essays, literature reviews and analyses (**Knows How**):	*n* = 2; 66%
	Demonstration of learning activities through case studies, discussion, applied projects and lab applications (**Shows**):	*n* = 6, 43%	Demonstration of learning activities through case studies, discussion, applied projects and lab applications (**Shows**):	*n* = 3, 100%
Structure of learning activities and assessments	Seek multiple perspectives (from different disciplines) on a topic:	*n* = 1, 7%	Seek multiple perspectives (from different disciplines) on a topic:	*n* = 1, 33%
	Think critically about the cause, and multiple causes, of a problem:	*n* = 3, 21%	Think critically about the cause, and multiple causes, of a problem:	*n* = 2, 66%
	Seek to understand recurring and broad patterns rather than singular events:	*n* = 3, 21%	Seek to understand recurring and broad patterns rather than singular events:	*n* = 2, 66%
	Identify and consider the multiple connected issues to a particular problem:	*n* = 3, 21%	Identify and consider the multiple connected issues to a particular problem:	*n* = 2, 66%
	Identify and consider the multiple causes and effects of a situation or action:	*n* = 2, 14%	Identify and consider the multiple causes and effects of a situation or action:	*n* = 1, 33%
	Understand that situations change over time, and so will solutions:	*n* = 2, 14%	Understand that situations change over time, and so will solutions:	*n* = 1, 33%
	Understand that multiple solutions or actions can affect one another:	*n* = 2, 14%	Understand that multiple solutions or actions can affect one another:	*n* = 2, 66%
Frameworks Guiding Teaching	None: The United Nations (UN) Brundtland definition of sustainable development (1987): The United Nations (UN) Sustainable Development Goals: The Food and Agriculture Organization (FAO) Sustainable Diets and Biodiversity proceedings (Burlingame, 2012): Mason & Lang's definition of sustainable diets: The FAO definition of Sustainable Food Systems: Sociological systems theory (Eakin et al., Norberg & Cumming, FSSD, etc.): Hamm & Bellows definition of Community Food Security: Kuhnlein's definition of Traditional Food Systems: Food Secure Canada's definition of Food Sovereignty: Plant‐based diets: Alkon & Agyemen's definition of Food Justice:			*n* = 1, 6% *n* = 5, 31% *n* = 5, 31% *n* = 6, 38% *n* = 6, 38% *n* = 3, 19% *n* = 1, 6% *n* = 1, 6% *n* = 1, 6% *n* = 4, 25% *n* = 4, 25% *n* = 1, 6%
Focus of SFS topics in teaching and average difficulty 1—lowest difficulty, 5—highest difficulty *based on 7 responses (across graduate and undergraduate courses)	Environmental sustainability (biodiversity): 3.43			
	Environmental sustainability (climate change): 2.86			
	Environmental sustainability (energy use): 3.14			
	Environmental sustainability (food waste): 1.86			
	Environmental sustainability (land use): 2.57			
	Environmental sustainability (water use): 2.43			
	Social sustainability (culture): 2.43			
	Social sustainability (economy): 3.71			
	Social sustainability (equity): 2.14			
	Social sustainability (gender): 2.43			
	Social sustainability (sovereignty): 2.57			
	Professional roles (sustainability/nutrition policies): 4.0			
	Professional roles (sustainability/nutrition programs): 3.43			
	Professional roles (meal planning, menu design): 3.14			
	Professional roles (sustainability‐informed dietetic curriculum): 4.14			

Most SFS courses occur in person and offer varying degrees of experiential and land‐based learning. The top three approaches to teaching across all courses were a combination of lectures, discussions, and community service learning. To promote systems thinking, educators most often opt for structured learning activities and assessments that encourage students to think critically about the cause and multiple causes of a problem, and to identify and consider the multiple connected issues related to a particular problem. Table 2 outlines the characteristics of SFS‐related undergraduate and graduate courses.

### 3.3 SFS Challenges and Opportunities in Dietetics Programs

Four overarching themes were identified through qualitative analysis: (1) limits of current SFS coverage (affecting future practice); (2) necessary support from departmental, institutional and professional levels; (3) students' needs and learning outcomes, and (4) examples of effective SFS teaching to guide, inspire and inform.

We integrate themes one and two in our reporting because they include interrelated barriers and supports, and for brevity. We present the limits of current SFS coverage (theme 1), including: a lack of coordinated, scaffolded learning; a minimum curriculum that is instructor‐dependent; and the need for pedagogy that supports effective learning, with the necessary supports from departments, institutions, and professional associations (theme 2). These supports include findings related to *what* SFS content should be included, *where* it should be placed in the curriculum, and *how* to deliver it effectively, which is woven into each section. Following this, considerations for the unique needs of today's students in our current context (theme 3) are reported, along with examples of effective practice (theme 4).

### 3.4 Coordinated, Scaffolded, Program‐Wide Learning

Education related to SFS is uneven across the country, with only approximately 50% of the undergraduate courses described in our study identified as *required* or *mandatory* for nutrition students. Despite widespread acceptance of the need for SFS education, *most people are thinking/appreciative of that nowadays …* [P23], participants recognized the limitation in SFS education as being isolated to one or two courses, noting a lack of departmental planning and coordination, or *weaving it in* [P1] across multiple courses. Participants [P2, P15] were critical of the current practice of teaching SFS like a *‘special topic’*, rather than embedded in what we know and teach. Lack of coordinated, scaffolded, program‐wide SFS coverage was a gap linked to the perception that programs were merely *scratching the surface* [P1] in SFS education and ‘*we cannot box it into one course…it needs to get into these other courses…so that we [have] people who are very conscious about sustainability right from the beginning*. [P1]. Educators were concerned about missed opportunities to reinforce and add depth across multiple courses and program years, and well‐planned scaffolding was recognized as the ideal.

Departmental support that moved towards intentional planning of SFS across the program was identified as a necessary support among educators. Suggested departmental activities included agreeing on a comprehensive definition of SFS and clear student learning outcomes, as well as conducting curriculum mapping exercises to identify what SFS concepts are taught, where they are taught in the curriculum, and to assess existing gaps. One participant [P8] suggested an *SFS advisor* role to review the educator's teaching content and identify specific places where SFS could be incorporated. Support from the profession would aid departments in this work and ensure consistency across accredited dietetics programs, and promote greater faculty buy‐in for scaffolded SFS education. The Integrated Competencies for Dietetic Education and Practice (ICDEP) were discussed as the most critical driver for curricular change. The ICDEPs provide the mandate for what is taught in Canadian dietetics programs and at present are perceived as a barrier for two reasons: (1) the lack of explicit, detailed SFS competency statements and measurable key performance indicators, and (2) the observation that *… Sustainability appears to be [positioned as] a specialty over an area that needs to be threaded into all of dietetics* [P15].

### 3.5 Minimum Curriculum Dependent on Instructor

Participants noted that the content and depth of SFS education are highly dependent on the individual instructor's readiness, initiative, expertise, and experience. Concerns, introduced above, about a gap in *a basic minimum* [P1] set of topics necessary to meet the ICDEP competencies, were also perceived to exacerbate the dependence on instructor readiness and increase preparation‐related time burden (e.g., no standard text to follow, insufficient time to research or participate in an SFS network). Both make SFS content vulnerable to a practice of *patch[ing] things here and there*, [P1] to create relevant teaching content.

Educators are grappling with how to define the core SFS knowledge and skills needed for entry‐level dietitians, often using diverse frameworks and resources (see Table 2). Respondents most frequently described gaps in their knowledge and experience related to Indigenous knowledge, interdisciplinary collaboration and networks, methods to calculate SFS impacts, and access to Canadian‐relevant content and data.

Support from the profession regarding how best to deliver SFS content beyond the ICDEPs was identified as a need among educators, including an interest in defined learning objectives and clear criteria to guide the selection and inclusion of SFS content. Respondents suggested a *sample curriculum in different packages* [P2] that includes practical, evidence‐based tools, such as case studies, infographics, resource banks, and videos, which align with ICDEP competencies. Such a resource package would enable educators to tailor SFS content according to available teaching time and context. Professional development for teaching SFS was also identified as helpful.

### 3.6 Beyond Minimum Curriculum: Pedagogy for Effective SFS Learning

Educators identified concerns with bridging theory to practice. A common issue in SFS teaching, particularly given the limited availability of practice‐based resources, was the concern among educators that students may struggle to apply SFS concepts effectively in various dietetic practice settings.

The educator‐dependent challenges described above were perceived as being exacerbated by limited class time, competing priorities within the curriculum, and general resource constraints for teaching. Common concerns related to a lack of funding for guest speakers (i.e., ethical honoraria to Indigenous community members), insufficient dedicated staff (i.e., community coordinators), and inadequate physical infrastructure (e.g., teaching kitchens, outdoor learning spaces). Participants described the importance of local community connections for teaching Indigenous food systems, underscoring the diversity and specificities of knowledge holders. Only one program described having a faculty member with expertise in Indigenous food systems, highlighting a notable gap in Canadian programs. Among educators, this expanded pedagogy was understood to be crucial for understanding complex food systems, fostering critical thinking and systems thinking, and developing practical skills; however, it was often perceived as under‐resourced and limited in most programs (see Table 2).

### 3.7 Students' SFS Needs and Learning Outcomes

Current students were perceived to be more aware and critically engaged with SFS issues: *Some of them, not all, are really great critical thinkers…already thinking about climate change…how to mitigate climate change through food systems. And they're…going above and beyond what is being taught to them and they're bringing that to classrooms and having discussions.* [P5]. This emphasized the importance of tailoring content to reflect students' existing knowledge and concerns (e.g., climate change, food literacy, food security), as well as skill level.

Unanimously, and despite occasional resistance from students less comfortable with a systems‐based approach, educators emphasized the need to develop students' systems thinking skills, which were viewed as critical for understanding complexity and moving beyond individualistic, reductionist/deficit‐based approaches to change. As one graduate educator [P2] remarked, *Students want to know how they can create change, that requires multi‐year, transformative and systems‐engaged pedagogy*. Several other educators described using ‘sustainability’ as a critical lens through which to develop systems thinking skills while exploring challenging and complex topics, such as food justice, anti‐oppression, health equity, and Indigenous food sovereignty.

### 3.8 Examples of Effective SFS Education

Participants provided insights into effective SFS education through success stories within their teaching, highlighting that [1]: university‐wide support and connections can catalyze impactful SFS teaching [2]; institutional, departmental and grant funding increases opportunities for experiential and diverse perspective learning; and [3] accreditation can provide structure to support course planning for SFS education.

University‐wide support and connections catalyze impactful SFS teaching. At one institution, interdisciplinary, cross‐campus connections between experts and practitioners interested in SFS translated into a dedicated course that was mandatory for dietetic students, open to students from other disciplines, and housed within the dietetics program. Access to campus experts co‐instructing the course helped address a key knowledge and experience gap among dietetic educators: *none of us felt like we had the expertise for this course, which was why that collaboration with people across campus was so key to the success…because we didn't feel that we had the training in that at all, or familiarity with research in areas of sustainability* [P3].

Institutional, departmental and grant funding increase opportunities for experiential and diverse perspective learning. Departmental funding for a lab instructor or coordinator position provided critical support for experiential learning, allowing students to extend their understanding of SFS beyond the classroom: *I think the lab component is useful because it gives the students time to practise some of these things, even if it's from theory. …The fact that there's a lab, and the lab really contributes* [to hands‐on learning] [P1]. Conversely, the recent loss of funding for a community coordinator position was devastating to one program: *Knowing that we have top level support, knowing that students are demanding it [experiential learning], but also knowing that just last month, we just got our last piece of [departmental] support cut, which is crazy*….*It's gone*. [P4]. A short‐term institutional grant to support guest lecturers and co‐instructors was another example of excellence: *So I was able to bring in speakers that I was able to record and then reuse throughout the course for those certain weeks and topics on Inuit food systems, Metis food systems, and three different First Nations. And leaders working on revitalizing their food systems, but from different areas of Canada.* [P5].

Accreditation has the potential to facilitate intentional course planning for SFS education. The accreditation requirements (including the ICDEPs) were perceived to foster high levels of organization to support the scaffolding of SFS coverage, encourage evidence‐based practice, and promote strong career preparation: *The other programs…they don't even know what the next person is teaching for the most part because they don't have to… [but dietetics programs have the] responsibility of being on their game because of accreditation…. And I don't see that level of integrity in my other colleagues…to coordinate [course content] with their colleagues is like an intention, but it doesn't actually usually happen.* [P4].

## 4 Discussion

This research is the first to explore how SFS concepts are taught in accredited dietetics programs in Canada, identifying challenges and supports required to ensure that Canadian dietitians are prepared to play leadership roles in facilitating healthy and sustainable dietary patterns and food environments. Building on previous research that demonstrates a movement towards SFS‐informed education in Canada [11, 35], these findings deepen our understanding of the Canadian context and provide specific recommendations relevant to dietetics education programs, universities and professional regulators.

Recommendation 1 Increase SFS Specificity within the Competency Standards

Including specific SFS concepts in the standards governing the content of Canadian accredited dietetics programs [27] facilitates the systemic integration of SFS education, impacting all programs. However, at present, the Canadian standards require more specificity [28]. Other areas of competence within the ICDEPs are articulated in more detailed ways, providing robust curricular guidance and indicating the level of appropriate depth. Examples of appropriate and adaptable content exist [29] and are influencing the degree and focus of SFS education within accredited dietetics programs in the United States [36].

Canadian dietetic competency standards comprise four statements and five practice indicators that focus on understanding Indigenous peoples and the role of Indigenous food systems [28]. This Canadian‐focused research highlighted the importance of Indigenous perspectives in SFS education, including opportunities for faculty and community leaders (including those with relevant food systems expertise but without recognized professional titles or designations) to support student learning. An example of teaching excellence was possible through a short‐term university grant that allowed one educator to provide ethical honoraria to Indigenous community members. Specifically, participants identified their experience and connections with Indigenous communities as *pairing well* [P27] (supporting their SFS learning and teaching), with a clear appreciation and respect for *Indigenous practices fall[ing] within SFS practices* [P27]. While it is essential that these competencies are not subsumed under an SFS umbrella, it is important to acknowledge that they are complementary to SFS competencies and must be prioritized, given Canada's commitments to the Truth and Reconciliation, which include actions directed at the Health and Education sectors [37]. Reconciliation activities in Canadian dietetic education and training programs have been described elsewhere [38] and highlight the importance of dietetic leadership. As a profession, we can move closer to the social ideals of ‘civic dietetics’, as described by Wilkins and colleagues [8], by increasing specificity in the Canadian dietetic competency standards and promoting greater consideration of diverse ways of knowing, while upholding social justice and equity as central tenets of SFS.

Recommendation 2 Develop Curriculum Resources and Tools for Consistent Training

Understanding and action for SFS is necessarily transdisciplinary [39]. In addition to updates to the ICDEPs, practice‐informed curricular guidance that reflects the needs of dietetic educators for diverse knowledge of SFS is needed to advance these efforts. The current lack of clear direction on what, where, and how to teach SFS in the curriculum was noted as a concern among educators and thought to contribute to uneven coverage across Canadian dietetic programs, reliance on individual faculty initiative, and a lack of standardized preparedness among graduates. In this study, 81% of participants were RDs, and 44% were part‐time educators or staff in non‐tenured (non‐permanent) positions. Considering the lack of standardized content and the threat of losing SFS educators on short‐term contracts, the current state of SFS education within dietetic programs is precarious. This link between curriculum quality and work precarity is well understood in university teaching literature [40, 41].

Respondents identified the need for an adaptable SFS curriculum and effective teaching tools. Regarding curriculum development, work is underway on a global SFS education framework, informed by scholars, educators and practitioners [2, 36, 42, 43]. Respondents suggested tools such as case studies, infographics, resource banks, and videos that align with ICDEP competencies. Previous work to compile and share lesson plans exists [44, 45], as do tested, online resources [46, 47], and resource banks [48, 49]. So, while resources abound, they exist scattered across many sources, amplifying the time‐consuming preparation barriers highlighted in this research.

Recommendation 3 Embed the SFS Curriculum Throughout Education and Training to Promote Systems‐Engaged Pedagogy

Currently, SFS concepts in accredited dietetics programs in Canada are mainly constrained to a single course (or ‘box’). A critical perspective emerging from the key informant interviews, and supported by sustainability education scholars [16] is that SFS should be ‘unboxed’—that is, not limited to a single box but rather planned and integrated strategically across multiple courses in upper and lower program years in a scaffolded approach. This systemic pedagogical shift would demonstrate to students that SFS is relevant to all areas of practice—not a specialty topic—and reinforce that this is how dietetics is—and should be—*practiced*.

‘Unboxing’ SFS was related to an explicit goal for many educators: to foster systems thinking and help students understand—and be prepared to address—the key challenges related to the root causes of health and power dynamics within food systems. This assertion is discussed by other scholars [19, 21] as creating opportunities to deepen engagement and learning over time, increasingly seeing systems and thinking critically about the integral connection between human health and the planet. Without intentional scaffolding and the systemic embedding of SFS education, graduates from these programs face multiple, system‐level complexity challenges, to which they may lack the skills, knowledge, or professional networks to navigate.

Recommendation 4 Signal Importance and Claim Space.

The positioning of SFS education matters. When the SFS curriculum includes a general program elective offered to university students across multiple disciplines, it signals that the content is optional. In contrast, experiential learning opportunities and course content dedicated to SFS that are mandatory and informed by diverse perspectives *within* a dietetics program, offer a clearer message to future professionals: *this is of utmost importance, and not only will you need to ‘Know’ and ‘Know How’, you will also be required to ‘Show’* [27] through specific demonstration of learning activities relevant to dietetic practice. Signalling the importance of SFS education by housing the learning within a dietetics program and making it mandatory for students requires programs to engage in planning and resource prioritization (e.g., time, money), which may be limited. However, it will ensure that what, where and how SFS is taught is aligned with the specific leadership needs and professional roles of those practicing dietetics in the 21st century.

### 4.1 Limitations

This research was conducted during a period of increased uncertainty among Canadian dietetic educators. Post‐pandemic university spending cutbacks, changes in the leadership and regulation of dietetic accreditation and review cycle of the ICDEPs may have contributed to an unusual level of discouragement among educators concerning Canadian dietetics. For program leads and educators, these factors consumed a significant amount of time and may have negatively impacted participation rates and the overall tone and type of responses. Furthermore, strict eligibility criteria, survey length and question branching limited the number of respondents who completed the entire questionnaire. No in‐depth interviews (no response) were conducted with French educators, resulting in a missed opportunity to gain valuable perspectives on cultural or linguistic differences in SFS education. Researcher positionality was a strength in the interpretation of the results, as researchers represented dietetic programs of varying sizes (enrollment), geography, and graduate‐level education opportunities. Insight into the diversity within Canadian dietetic programs was critical in the development of recommendations for SFS education. There were no professional conflicts of interest that may have influenced interviewee responses.

## 5 Conclusion

As climate change continues to exacerbate food security and public health risks, dietitians must be prepared to lead in the transition toward healthy, sustainable, fair, and resilient food systems. Embedding SFS into dietetics education is necessary for equipping future professionals with the knowledge and skills necessary to promote healthy and sustainable dietary patterns and food environments. Dietetic program directors and nutrition faculty can work strategically to ‘unbox’ SFS from a single course, embedding SFS throughout education and training to prepare graduates and signal the importance of systems‐informed practice. Professional regulators can enhance the specificity of SFS within competency standards and collaborate with researchers to develop curriculum resources and tools that support consistency in SFS education and training across diverse programs and institutions. University administrators can play a crucial role in signalling the importance of SFS education through strategic leadership and by allocating support for research, teaching, and interdisciplinary collaboration, which are necessary to bring together diverse perspectives to support student learning. Addressing the barriers identified in this research will further enhance the impact of sustainability education in dietetics programs. Findings can be used to support dietetics programs in planning for more intentional, multi‐year, transformative, and systems‐engaged SFS pedagogy.

## Author Contributions

J.W., L.C., and T.C. contributed equally to all stages of the research and writing processes. N.K. and J.S. contributed to the initial data analyses, and N.K. contributed to the introduction through a detailed literature review.

## Ethics Statement

Ethics approval was obtained through Toronto Metropolitan University (REB 2022‐316), Acadia University (REB 22‐41), and St. Francis Xavier University (REB 26076). This manuscript does not use language that is stigmatizing or prejudiced when referring to study participants.

## Conflicts of Interest

The authors declare no conflicts of interest.
